# A pharmacogenetic intervention for the improvement of the safety profile of antipsychotic treatments

**DOI:** 10.1038/s41398-019-0511-9

**Published:** 2019-07-25

**Authors:** Maria J. Arranz, Alex Gonzalez-Rodriguez, Josefina Perez-Blanco, Rafael Penadés, Blanca Gutierrez, Laura Ibañez, Barbara Arias, Mercè Brunet, Jorge Cervilla, Juliana Salazar, Rosa Catalan

**Affiliations:** 1 Fundació Docència i Recerca Mútua Terrassa, Terrassa, Spain; 2 Centro de Investigación en Red de Salud Mental, CIBERSAM, Madrid, Spain; 3 Department of Mental Health, Parc Taulí University Hospital Sabadell, Barcelona, Spain; 4 Department of Psychiatry, Hospital de la Santa Creu i Sant Pau, Barcelona, Spain; 5 Barcelona Clinic Schizophrenia Unit (BCSU), Neurosciences Institute, Hospital Clinic of Barcelona, University of Barcelona, IDIBAPS, Barcelona, Spain; 6 Department of Psychiatry, University of Granada, Granada, Spain; 7 Department of Biologia Evolutiva, Ecologia i Ciències Ambientals, Facultat de Biologia, Institut de Biomedicina de la Universitat de Barcelona (IBUB), Barcelona, Spain; 8 Pharmacology and Toxicology Unit, Department of Biochemistry and Molecular Genetics, Hospital Clinic, Barcelona, Spain; 9 Genetics Department, Hospital de la Santa Creu i Sant Pau, Barcelona, Spain

**Keywords:** Personalized medicine, Clinical genetics

## Abstract

Antipsychotic drugs fail to achieve adequate response in 30–50% of treated patients and about 50% of them develop severe and lasting side effects. Treatment failure results in poorer prognosis with devastating repercussions for the patients, carers and broader society. Our study evaluated the clinical benefits of a pharmacogenetic intervention for the personalisation of antipsychotic treatment. Pharmacogenetic information in key CYP polymorphisms was used to adjust clinical doses in a group of patients who started or switched treatment with antipsychotic drugs (PharmG+, *N* = 123), and their results were compared with those of a group of patients treated following existing clinical guides (PharmG−, *N* = 167). There was no evidence of significant differences in side effects between the two arms. Although patients who had their antipsychotic dose adjusted according to CYPs polymorphisms (PharmG+) had a bigger reduction in side effects than those treated as usual (PharmG−), the difference was not statistically significant (*p* > 0.05 for all comparisons). However, PharmG+ patients treated with CYP2D6 substrates that were carriers of CYP2D6 UMs or PMs variants showed a significantly higher improvement in global, psychic and other UKU side effects than PharmG− patients (*p* = 0.02, *p* = 0.05 and *p* = 0.01, respectively). PharmG+ clozapine treated patients with CYP1A2 or CYP2C19 UM and PMs variants also showed higher reductions in UKU scores than PharmG− clozapine patients in general. However, those differences were not statistically significant. Pharmacogenetic interventions may improve the safety of antipsychotic treatments by reducing associated side effects. This intervention may be particularly useful when considering treatment with antipsychotics with one major metabolic pathway, and therefore more susceptible to be affected by functional variants of CYP enzymes.

## Introduction

Antipsychotic drugs are widely used for the treatment of severe mental illness including schizophrenia, bipolar disorder and major depression. However, 30–50% of treated patients fail to respond adequately and about 50% of them develop severe and lasting side effects. Treatment failure results in poorer prognosis with devastating repercussions for the patients, carers and broader society. The reasons for treatment failure are unclear and ‘trial and error' naturalistic strategies are used to select drug type and clinical doses for each patient.

Growing evidence indicates that genetic factors play a critical role in determining the clinical outcome of antipsychotic treatment^1,2^. Several genetic polymorphisms have been identified in drug metabolic enzymes that influence metabolising rates and clinical outcome. Cytochrome P450 enzymes, responsible for the biotransformation of more than 85% of existing drugs, may contain genetic variants that result in poor, intermediate, extensive or ultra-rapid metabolic rates^3,4^. Individuals with slow metabolising CYP variants are prone to adverse reactions, and require lower doses, whilst patients with ultra-rapid metabolising CYP variants require higher doses to obtain therapeutic response. CYPs are responsible for the biotransformation of widely used antipsychotics such as clozapine (CYP1A2 and CYP2C19), olanzapine (CYP1A2), risperidone and haloperidol (CYP2D6), quetiapine and ziprasidone (CYP3A5) amongst others^5,6^. The direct correlation between presence of slow metabolising CYP variants and antipsychotic-induced adverse reactions is well documented, although their influence on the level of efficacy in less clear^7,8^. It has been suggested that the adjustment of clinical doses according to the CYP genetic profile (presence of functional polymorphisms) of patients has the potential to significantly improve the efficacy (15%) and safety (25% reduction in side effects) of pharmacological treatments^9^.

There are several commercially available pharmacogenetic tests that interrogate CYP functional polymorphisms, which can be potentially useful for the personalisation of antipsychotic drugs^10^. However, their use in clinical settings is minimal. Recent studies have proven the benefits of using pharmacogenetic information for the personalisation of treatment in psychiatry. CYPs, UGTs and multidrug resistance 1 (MDR1) genotyping for antidepressant dose adjustment improves remission^11^. The use of commercial tests interrogating key CYP polymorphisms and serotonin genes resulted in improved response to antidepressants^12–15^. A recent study in elderly patients treated with antidepressants and antipsychotics according to their pharmacogenetic profile had a significant decrease in hospitalisations and emergency department visits^16^. Pharmacogenetic-guided prescription of antidepressants also resulted in improved adherence and reduced pharmacy costs^17,18^. However, most studies performed to date have focused on antidepressant medications. It is not yet sufficiently established the clinical and economic benefits of introducing pharmacogenetic interventions (PIs) for the improvement of antipsychotic treatment. In addition, rapid testing protocols that deliver clear recommendations on antipsychotic choice and dose are required to increase the implementation of pharmacogenetics in clinical practice.

The aim of this study was to evaluate the clinical benefits of a PI for the personalisation of antipsychotic treatment. Pharmacogenetic information in key CYP polymorphisms was used to adjust clinical doses in a group of patients who started or switched treatment with antipsychotic drugs, and their results were compared with those of a group of patients treated following existing clinical guides. We obtained encouraging results indicating that pharmacogenetic-guided antipsychotic prescription may increase the safety of antipsychotic treatments.

## Materials and methods

### Description of sample

Two hundred and ninety patients with a diagnosis of schizophrenia, schizoaffective or delusional disorders (according to DSM-5) and requiring antipsychotic treatment completed the study. Patients were recruited in the mental health wards of three hospitals (Hospital Clínic and Hospital de la Santa Creu i Sant Pau, Barcelona; Hospital Universitario, Granada) and were mostly outpatients. Patients were randomly selected for PI (PharmG+ arm) or treatment as usual (PharmG− arm). Randomisation was conducted using a web programme (www.randomiser.org). *Pharmacogenetic arm (PharmG+):* 123 patients were genotyped using a pharmacogenetic test at the beginning of their treatment with a new antipsychotic and clinical doses were adjusted accordingly when required (PharmG+). *Naturalistic arm (PharmG*−*):* 167 patients were treated as usual following standard clinical practices. This sample has a statistical power > 95% to detect genetic associations with a medium effect size (*f* = 0.25, *α* error = 0.05, calculated with GPower version 3.0.10).

### Response assessment

The positive and negative syndrome scale for schizophrenia (PANSS^19^) and the UKU-side effect rating scale (UKU-SERS^20^) scores were obtained in all patients at the beginning and after 12 weeks to assess the efficacy and safety of the treatment. PANSS and UKU values were scored by trained psychiatrists who were blind to the patients' pharmacogenetic arm. Within each participating hospital, the same psychiatrist evaluated the pre and post treatment patient status for both arms. Adherence was confirmed by plasma levels at week 12 of treatment. Table 1 summarises the clinical and demographic data of the study samples. All participants gave informed consent to the study, which was approved by the local ethics committees.

**Table 1:** Clinical and demographic data of the study samples

	Study arm		Totals
	PharmG+ (*N* = 123)	PharmG− (*N* = 167)	Total (*N* = 290)
Gender			
Male	63 (51.2%)	94 (56.3%)	157 (54.1%)
Female	60 (48.8%)	73 (43.7%)	133 (45.9%)
Age			
Mean (SD) (years)	46.11 ± 13.7	48.68 ± 13.46	
Diagnosis			
Schizophrenia	86%	69%	76%
Schizoaffective	5%	4%	4%
Delusional disorder	9%	27%	20%
Treatment			
Drug			
Clozapine	43 (35%)	88 (52.7%)	131 (45.2%)
Risperidone	16 (13%)	20 (12%)	36 (12.4%)
Olanzapine	25 (20.3%)	14 (8.4%)	39 (13.4%)
Paliperidone	16 (13%)	22 (13%)	38 (13.1%)
Aripiprazole	7 (5.7%)	13 (7.8%)	20 (6.9%)
Quetiapine	11 (8.9%)	5 (3%)	16 (5.5%)
Ziprasidone	1 (0.8%)	2 (1.2%)	3 (1%)
Trifluoperazine	1 (0.8%)	1 (0.6%)	2 (0.7%)
Haloperidol	1 (0.8%)	1 (0.6%)	2 (0.7%)
Asenapine	1 (0.8%)	1 (0.6%)	2 (0.7%)
Pimozide	1 (0.8%)	0	1 (0.3%)
Dose (olanzapine equivalent)			
Average dose (SD)	11.57 ± 7.28	10.65 ± 5.70	
PANSS basal scores			
Mean (SD)	99.23 ± 18.08	91.55 ± 17.55	
UKU basal scores			
Mean (SD)	9.15 ± 5.98	6.83 ± 5.40	

### Biological samples

Whole blood samples were collected from all participants at the time of recruitment. DNA was obtained using commercial kits (QIAmp DNA mini Kit, Qiagen) within hours of blood extraction and was immediately sent for pharmacogenetic testing (PharmaG+ patients) or stored at −80 °C until required.

### Pharmacogenetic intervention

A commercial pharmacogenetic test (Brainchip, Brainco, Bilbao, Spain) was used to characterise common and functional polymorphisms in CYP1A2, CYP2D6, CYP2C19 and CYP3A5 enzymes, the main metabolic pathways of currently available antipsychotics. Table 2 summarises the list of key CYP polymorphisms genotyped that included the most frequently detected in Caucasian populations^21^. A DNA sample from PharmG+ patients was obtained at the time of initiating or changing antipsychotic treatment and sent to Brainco for characterisation with the Brainchip array. Results were returned within 1–2 weeks of extraction to the responsible clinician, and dose adjustments, if necessary, were performed according to the study protocol. Patients were maintained on antipsychotic monotherapy during the duration of the study.

**Table 2:** Key CYP polymorphisms genotyped in the samples

Gene	Alleles genotyped	Known function
CYP1A2	*1	Normal
	*1F	Higher inducibility
CYP2C19	*1	Normal
	*2	No activity
	*3	No activity
	*4	No activity
	*8	Decreased activity
CYP2D6	*1	Normal
	*2	Normal
	*3	No activity
	*4	No activity
	*5	No activity
	*6	No activity
	*9	Decreased activity
	*10	Decreased activity
	*35	Normal
	*41	Decreased
	*2×N	Increased activity
	*4×N	Increased activity
CYP3A5	*1	Normal
	*3	Reduced activity

### Dose adjustment protocol

Patients were recruited when starting treatment with an antipsychotic or when a change in their antipsychotic treatment was required. All patients (PharmG+ and PharmG−) were informed of the study and were blind to the PI. No pharmacogenetic information was provided to the patients, nor were they told if changes in their medication had been required as a result. At the time of the study, there were no clinical guidelines on the level of dose adjustment required for patients with CYP functional polymorphisms. The clinical recommendations (dose adjustments) used in the study were decided by the clinical teams at the beginning of the project and are summarised in Table 3. Doses were adjusted according to main metabolic pathways:^5,6^ clozapine doses were adjusted according to the genotypic variants observed in CYP1A2 and CYP2C19; olanzapine doses were adjusted according to CYP1A2 polymorphisms; risperidone, aripiprazole, haloperidol, pimozide and trifluoperazine doses were adjusted according to CYP2D6 polymorphisms; finally, quetiapine and ziprasidone doses were adjusted according to CYP3A5 genetic variants. Paliperidone and asenapine doses were not adjusted unless they were given in combination with a second antipsychotic, in which case the second antipsychotic was used as a guide for adjustment. According to this protocol, *N* = 55 PharmG+ patients required dose adjustments of the antipsychotic drug received. Despite adjustments, clinical doses remained within recommended ranges in both arms.

**Table 3:** Recommended adjustment of clinical doses for patients undergoing pharmacogenetic intervention (PharmG+)

Antipsychotic	Standard clinical dose	CYP1A2			CYP2D6			CYP2C19		CYP3A5		
		PM	IM	UM	PM	IM	UM	PM	IM	PM	IM	UM
Clozapine	150–900 mg/day	<25–50%	NC	>25–30%				<25–30%	NC			
Olanzapine	7.5–30 mg/day	<25–50%	NC	>25–30%								
Risperidone	3–12 mg/day				<25–30%	Gradual	>25–30%					
Haloperidol	10–20 mg/day				<50%	<10–20%	>25–30%					
Aripiprazole	10–30 mg/day				<25–50%	Gradual	>25–30%					
Quetiapine	300–750 mg/day									<25–50%	<10–20%	>25–30%
Ziprasidone	80–160 mg/day									<5–50%	<10–20%	>25–30%

Gradual: 5–10% reduction every 3 days (up to 30% reduction) depending on side effects

### Genotypic characterisation of samples

All patients (PharmG+ and PharmG−, *N* = 290) were genotyped for the key CYP functional polymorphisms included in the pharmacogenetic array using iPlex^®^ Gold chemistry and the MassARRAY platform (CEGEN-PRB2-ISCIII, University of Santiago de Compostela, Spain) and TaqMan probes and technology for the CNVs (University of Granada). High-throughput genotyping results coincided with those obtained with the commercial array in overlapping samples and polymorphisms.

### Statistical analyses

Linear regression models to assess the influence of the PI were calculated considering level of response and side effects improvement as dependent variables. The PI (study arm PharmG+ or PharmG−) was included as a predictor variable and drug, gender, dose (olanzapine equivalent), age of patients and PI as additional covariables. All analyses were performed using the statistic package SPSS (version 22, IBM).

## Results

Tables 4 and 5 summarise the results of the statistical analyses. The CYP minor allele frequencies observed agreed with those described in European populations^21^. Despite randomisation during the recruitment, differences were observed in the distribution of treatments (*p* = 0.01) and in the severity of symptoms (*p* = 0.002) between arms (see Table 1). To minimise these differences, the PANSS or UKU score differences (basal scores − scores at 12 weeks of treatment) were considered as the dependent variables. In addition, treatment (antipsychotic drug) was one of the covariates considered in the regression model.

**Table 4:** Summary of statistical analyses in all samples

Clinical variable	All patients (*N* = 290)		Clozapine treated patients (*N* = 131)		Patients treated with CYP2D6 substrates (*N* = 61)	
	PharmG+ (*N* = 123)	PharmG− (*N* = 167)	PharmG+ (*N* = 43)	PharmG− (*N* = 88)	PharmG+ (*N* = 26)	PharmG− (*N* = 35)
Difference in PANSS average	26.81 + 1.3	26.60 + 1	29.36 + 2.3	26.0 + 1.4	24.33 + 3.0	26.47 + 2.0
Model χ2 (*p* value)	6.72 (0.24)		8.54 (0.08)		2.90 (0.72)	
PI Wald (*p* value)	0.04 (0.84)		1.32 (0.25)		0.34 (0.56)	
Difference in UKU scores	3.62 ± 0.66	3 ± 0.56	3.81 ± 1.10	2.67 ± 1.03	4.04 ± 1.34	2.69 ± 1.11
Model χ2 (*p* value)	10.19 (0.07)		5.67 (0.23)		2.79 (0.73)	
PI Wald (*p* value)	0.40 (0.53)		0.002 (0.97)		0.38 (0.54)	
Difference in psychic SE	2.02 ± 0.36	1.26 ± 0.30	1.84 ± 0.63	1.38 ± 0.52	1.60 ± 0.72	0.53 ± 0.53
Model χ2 (*p* value)	10.38 (0.07)		3.26 (0.52)		7.71 (0.17)	
PI Wald (*p* value)	1.53 (0.22)		0.03 (0.86)		0.64 (0.42)	
Difference in neurological SE	0.87 ± 0.20	0.94 ± 0.25	0.98 ± 0.32	0.54 ± 0.51	1.04 ± 0.48	1.25 ± 0.56
Model χ2 (*p* value)	12.49 (0.03)		12.65 (0.01)		2.01 (0.85)	
PI Wald (*p* value)	<0.01 (0.99)		0.01 (0.91)		0.08 (0.78)	
Difference autonomic SE	0.32 ± 0.26	0.17 ± 0.12	0.16 ± 0.51	0.37 ± 0.37	0.71 ± 0.45	0.28 ± 0.16
Model χ2 (*p* value)	4.09 (0.54)		4.42 (0.35)		10.03 (0.07)	
PI Wald (*p* value)	0.35 (0.56)		0.47 (0.49)		0.93 (0.33)	
Difference in other side effects	0.54 ± 0.24	0.52 ± 0.23	0.93 ± 0.30	0.38 ± 0.41	0.56 ± 0.30	0.44 ± 0.29
Model χ2 (*p* value)	9.67 (0.09)		2.53 (0.64)		2.45 (0.78)	
PI Wald (*p* value)	0.003 (0.95)		1.32 (0.25)		0.07 (0.79)	

*PI* pharmacogenetic intervention, *SE* side effects

**Table 5:** Summary of statistical analyses in individuals with functional variants in relevant CYPs

Clinical variable	All patients (*N* = 155)		Clozapine treated patients (*N* = 68)		Patients treated with CYP2D6 substrates (*N* = 41)	
	PharmG+ (*N* = 79)	PharmG− (*N* = 76)	PharmG+ (*N* = 30)	PharmG− (*N* = 38)	PharmG+ (*N* = 21)	PharmG− (*N* = 20)
Difference in PANSS average	26.88 + 1.4	25.92 + 1.3	30.10 + 3	24.74 + 2	26.58 + 3.5	28.63 + 3.7
Model χ2 (*p* value)	1.68 (0.89)		5.19 (0.27)		12.88 (0.03)	
PI Wald (*p* value)	0.16 (0.69)		1.78 (0.18)		1.03 (0.31)	
Difference in UKU scores	4.20 ± 0.76	3.01 ± 0.66	4.24 ± 1.39	3.08 ± 1.25	6.92 ± 2.09	1.27 ± 1.78
Model χ2 (*p* value)	8.80 (0.12)		3 (0.56)		8.20 (0.15)	
PI Wald (*p* value)	1.23 (0.27)		0.02 (0.89)		5.56 (0.02)	
Difference in psychic SE	2.40 ± 0.41	1.21 ± 0.37	2.14 ± 0.82	1.42 ± 0.70	2.50 ± 1.14	0.53 ± 0.77
Model χ2 (*p* value)	10.90 (0.05)		1.20 (0.88)		10.83 (0.06)	
PI Wald (*p* value)	3.30 (0.07)		0.14 (0.71)		3.96 (0.05)	
Difference in neurological SE	0.99 ± 0.24	1.08 ± 0.26	1.24 ± 0.40	0.67 ± 0.67	1.67 ± 0.64	0.53 ± 0.76
Model χ2 (*p* value)	11.27 (0.05)		15.91 (0.003)		3.63 (0.60)	
PI Wald (*p* value)	0.01 (0.91)		0.03 (0.87)		1.67 (0.20)	
Difference autonomic SE	0.31 ± 0.29	0.20 ± 0.13	0.07 ± 0.64	0.75 ± 0.41	1.33 ± 0.91	0.13 ± 0.13
Model χ2 (*p* value)	3.04 (0.69)		2.14 (0.71)		5.13 (0.40)	
PI Wald (*p* value)	0.22 (0.64)		0.66 (0.42)		1.29 (0.26)	
Difference in other side effects	0.59 ± 0.28	0.45 ± 0.29	0.86 ± 0.39	0.25 ± 0.74	1.08 ± 0.57	−0.3 ± 0.41
Model χ2 (*p* value)	7.39 (0.19)		6.20 (0.19)		7.99 (0.16)	
PI Wald (*p* value)	0.10 (0.75)		0.08 (0.78)		6.19 (0.01)	

*PI* pharmacogenetic intervention, *SE* side effects

No significant differences in variation in PANSS scores were observed between PharmG+ (26.81 ± 1.3 score reduction after 12 weeks, on average) and PharmG− patients (26.6 ± 1 score reduction after 12 weeks, on average) (see Table 4). Linear model regression considering PI (PharmG+ or PharmG− arm), age, gender, dose (olanzapine equivalent) and drug as covariates confirmed this observation (model χ2 = 6.72, *df* = 5, *p* = 0.24, PI Wald coefficient = 0.04, *p* = 0.84). Similarly, no significant differences in PANSS scores improvement were observed between PharmG+ and PharmG− patients treated with clozapine (29.36 ± 2.3 vs 26 ± 1.4, respectively; model χ2 = 8.54, *df* = 4, *p* = 0.08, Wald coefficient for PI = 1.32, *p* = 0.25). Analyses within the subgroup of patients treated with drugs mainly metabolised by the enzyme CYP2D6 (risperidone, haloperidol, aripiprazole, pimozide and trifluoperazine) also showed no differences with PharmG+ and PharmG− patients showing similar variations in PANSS scores (24.33 ± 3 vs 26.47 ± 2). This difference was not statistically significant (χ2 = 2.90, *df* = 5, *p* = 0.72 and Wald = 0.34, *p* = 0.56 for model and PI, respectively). When analysing patients with functional variants in CYP1A2, CYP2D6 and/or CYP2C19 (*N* = 155), no differences were observed between PharmG+ and PharmG− patients in PANSS improvement, nor was it observed in the subgroups of patients treated with clozapine (*p* > 0.05 for all comparisons, see Table 5). However, there was a significant difference when analysing PANSS score improvement between PharmG+ patients (26.6 ± 3.5) and PharmG− patients (28.63 ± 3.7) treated with CYP2D6 substrates. This significant difference was mainly caused by patients' gender (Wald = 6.25, *p* = 0.01) and dose (Wald = 6.25, *p* = 0.01) and not by the PI (Wald = 1.03, *p* = 0.31).

Analyses of improvement in side effects after 12 weeks of treatment (UKU basal scores − UKU scores after 12 weeks) revealed a slightly higher improvement (bigger difference in UKU score reduction) in PharmG+ than in PharmG− patients in general, especially in the UKU psychic subscales (Table 4). However, the contribution of the PI to these improvements was not statistically significant (*p* > 0.05 for all comparisons). Analyses of patients with functional variants in CYP1A2, CYP2D6 or CYP2C19 revealed clearer improvements in the profile of side effects in the PharmG+ patients in comparison to PharmG− patients. Psychic side effects were further reduced in PharmG+ patients (2.40 ± 0.41) in comparison to PharmG− patients (1.21 ± 0.37; χ2 = 10.90, *p* = 0.05 and Wald = 3.30, *p* = 0.07 for model and PI, respectively). The PI was significantly associated to improvements in total (6.92 improvement in PharmG+ vs 1.27 in PharmG−, Wald = 5.56, *p* = 0.02), psychic (2.50 vs 0.53 improvement, Wald = 3.96, *p* = 0.05) and other UKU side effects (1.08 vs −0.3, Wald = 6.19, *p* = 0.01) in the subgroup of patients treated with CYP2D6 substrates (see Table 5).

## Discussion

Despite growing evidence showing the influence of genetic factors on antipsychotic treatment efficacy, pharmacogenetic information is rarely used in clinical settings for the personalisation of treatment. Several studies have shown that most clinicians and patients are in favour of PIs^22–24^. However, the scarce evidence on the clinical and economic benefits of PIs in psychiatry hamper their implementation in clinical practice. In this study, we investigated the clinical benefits of using genetic information in CYP metabolising enzymes for the adjustment of clinical doses of a variety of commonly used antipsychotics and compared the results with those of patients treated as usual.

Several commercial pharmacogenetic tests are available for use in psychiatry^25^. Most of them interrogate key functional polymorphisms in drug metabolising enzymes. However, several of these tests include other polymorphisms of unclear clinical value. We used a pharmacogenetic test that gives detailed information on CYPs functional polymorphisms. CYPs genotypes were used for dose adjustments, whilst the additional polymorphisms genotyped by the test were not considered for the intervention. This alternative information should not be considered until confirmation of their relevance is obtained, as it may hamper the clinical value and implementation of pharmacogenetic tests^26^. In the case of antipsychotics, the most robust results are those that associate CYP functional polymorphisms with adverse reactions^7^, whereas other polymorphisms in dynamic genes have been also proven to be useful for the improvement of antidepressant medication^27^.

Our results showed that a PI consisting in adjustments of clinical doses in patients with CYP alterations did not have a significant influence on the level of efficacy of antipsychotic treatments (*p* > 0.05 in all comparisons). This was somehow expected. Given the complex pharmacodynamic profile on antipsychotic drugs, treatment success may be influenced not only by drug pharmacokinetics, but also by pharmacodynamic interactions with the many receptors targeted by currently available antipsychotic drugs. Therefore, the possible influence of altered metabolism on drug efficacy may be diluted. Previous genetic association studies have failed to find a clear association between CYPs functional polymorphisms and antipsychotic treatment response^8^.

Nevertheless, strong evidence supports the influence of CYPs variants on the development of drug-induced adverse reactions^7,22,28–30^. Interestingly, we found that patients who had their antipsychotic dose adjusted according to key polymorphisms (PharmG+) had a bigger reduction in side effects than those treated as usual (PharmG−). This finding was not statistically significant when investigating all the participants in the study. However, when looking at the subgroup of patients who had functional variants in the CYPs responsible for the metabolism of their prescribed antipsychotic, the results were clearer. Our PI was particularly useful in the reduction of psychic symptoms (*p* = 0.07) in patients with CYPs functional variants. The subgroup of patients treated with CYP2D6 substrates (risperidone, haloperidol, aripiprazole, pimozide and trifluoperazine) that were carriers of CYP2D6 UMs or PMs variants clearly benefited from the intervention: model regression analyses showed that the PI resulted in a significant improvement in global, psychic and other UKU side effects (*p* = 0.02, *p* = 0.05 and *p* = 0.01, respectively). PharmG+ clozapine treated patients also showed higher reductions in UKU scores than PharmG− clozapine patients in general. However, those differences were not statistically significant, even when considering patients with CYP1A2 or CYP2C19 UM and PMs variants (*p* > 0.05 in all comparisons). These results suggest that a PI would be particularly useful when considering treatment with antipsychotic drugs with one main CYP pathway, where the influence of UMs and PMs variants should be more evident.

Previous studies on the benefits of PIs in psychiatry have focused mainly on antidepressant treatments. Several studies have proven the clinical value of a commercial array combining genetic information in metabolic enzymes (CYP1A2, CYP2D6 and CYP2C19) and antidepressant targets (serotonin transporter, *SLC6A4* and serotonin receptor 2A, *HTR2A*). Patients whose antidepressant drug and dose were selected according to the array polymorphisms showed better symptom improvement than patients treated as usual^12,13^. Similar results were found in a recent study in Spanish patients, also using a commercial kit that included CYPs, multidrug resistance 1 (MDR1 o ABCB1) and other target variants^1^. A study using the same commercial kit in a retrospective study on psychiatric patients receiving a variety of psychotropic drugs observed that those patients who had followed the test recommendations showed better treatment response^31^. A recent study in which clinicians were provided pharmacogenetic information based on CYP2D6 and CYP2C19 variants to adjust antipsychotic and antidepressant treatments in 80 psychiatric patients suggested a favourable opinion on the outcome of the intervention, although no control group was considered in the study^32^. Interestingly, Herbid et al. showed in a previous study that the adjustment of clinical doses according to CYP2D6 and CYP2C19 polymorphisms resulted in a reduction of treatment costs in schizophrenia patients showing poor or ultra-rapid metabolism^33^. However, no previous study has focused on the prospective evaluation of clinical improvement when using pharmacogenetics for the adjustment of antipsychotic treatments. To our knowledge, our findings are the first evidence of the clinical benefits obtained by a pharmacogenetic selection of antipsychotic clinical doses in comparison to naturalistic practices.

Our study has several limitations. Despite randomisation during recruitment, antipsychotic treatments and severity of symptoms were not evenly distributed in the study arms (PharmG+ and PharmG−). To minimise the effect of these differences on results, both factors (antipsychotic treatment and basal symptom severity) were included as covariates in the analyses. Sample size was dimed sufficient for the study aims. However, the limited number of patients carrying CYP functional polymorphisms did not allow the investigation of functional groups (UMs and PMs separately). In addition, the variety of treatments used in the study hampered the investigation of individual drugs. Nevertheless, we performed analyses on the subgroups of patients carrying CYPs functional variants (either UMs or PMs), and investigated the two largest antipsychotic groups within the study (clozapine and CYP2D6 substrates). Finally, a CYP2D6*17 polymorphism with reduced activity was not investigated in our study as it was not included in the array used for the PI. However, the reported frequency of this allele in the Spanish population is very low (0.0093) and should not greatly influence the results^34^.

In summary, although we found no evidence of greater efficacy, a PI may improve the safety profile of antipsychotic treatments in patients presenting poor or ultra-rapid CYP variants. The PI described in our study may be particularly useful when considering treatment with antipsychotics with one major metabolic pathway, and therefore more susceptible to be affected by functional variants of CYP metabolising enzymes.
